# On the Causes of Hæmorrhage during Pregnancy

**Published:** 1894-05-05

**Authors:** James Oliver

**Affiliations:** Physician to the Hospital for Women, London


### MEDICAL PROGRESS AND HOSPITAL CLINICS.

[*The Editor will be glad to receive offers of co-operation and contributions from members of the profession. All letters should be addressed to* THE EDITOR, THE LODGE, PORCHESTER SQUARE, LONDON, W.]

### ON THE CAUSES OF HÆMORRHAGE DURING PREGNANCY.

By JAMES OLIVER, M.D., F.L.S., F.R.S. (Edin.), Physician to the Hospital for Women, London.

In the present paper we shall deal only with the occurrence of hæmorrhage during the evolution of uterine pregnancy. In order, however, that we may have a clear conception of the manner in which the various causes of this untoward event exert their influence, it is befitting that we should recall those changes which it is alleged take place in the ovum and uterus after fecundation, and that we should note especially the probable period at which these changes take place.

It is stated that the fecundated ovum when it arrives in the uterus is five or six times its primitive or unfertilised size, and that the mucus lining of the uterus is then swollen and plicated. In a well-marked depression the ovum is arrested, and soon thereafter, as early even as the twelfth day of pregnancy, it is said the ovum becomes completely enveloped by the decidua reflexa, a membrane which is structually continuous with the decidua serotina, or that portion of the mucus membrane which participates in the formation of the true placenta. Through the medium of the decidua serotina, the decidua vera, or that portion of the mucus substance which covers the non-placental part of the uterus, is in its turn continuous with the decidua reflexa. The mode of origin of the reflexa has not yet been determined. It is generally stated, however, that eventually the decidua reflexa becomes united to the vera, but Minot affirms that the reflexa disappears during the fifth month of pregnancy by degeneration and absorption.

"By the time that the ovum," says Quain,* "becomes fixed in that part of the uterus which it is to occupy during the subsequent period of its intra-uterine life, a great change takes place in the nature of the external covering of the ovum by its conversion into a new membrane which acquires more or less of a composite villous structure." These villi, which at first are non-vascular, fit into the folds of the uterine epithelium and fulfil not only the function of fixing the ovum but of nourishing it. Towards the end of the third week of pregnancy these villi of the chorion become vascular through the agency of the allantois which, rapidly developing, thrusts its blood vessels into these processes indiscriminately, so that at this period the egg is practically covered by placenta. We are still ignorant of the cause which determines the location of the true placenta—a structure which is definable about the tenth week of pregnancy—but after the sixth or eighth week those villi, which are not destined to participate in the formation of this structure, atrophy and finally disappear.

As soon as the chorionic villi become vascular well-marked changes take place in the lining membrane of the uterus, but more especially in that portion which is to receive the placental villi. At this spot where the so-called decidua serotina develops the mucous and sub-mucous layers of the uterus become exceedingly spongy, and the chorionic villi increasing in size and becoming more branched insinuate themselves into this structure which has been prepared for their implantation. According to Turner the uterine vessels which enter into the formation of the maternal portion of the placenta lose their capillary form and become expanded into large, freely communicating sinuses. In these sinuses the fœtal villi hang, and it is only here and there that these structures are actually attached to the walls of the sinuses by means of trabeculæ, which pass from the serotina to the fœtal villi. In the majority of cases it is probable that the hæmorrhage which comes from the interior of the uterus during pregnancy is due to the rupture of one or more of these vascular sinuses, and that it seldom is derived from the fœtal villi, the vessels of which retain their capillary form.

The growing ends of the chorionic villi are sensitive to slight pressure, and in order that these processes may form with the spongy portion of the uterus a compact structure, it is requisite that their growth should, to a certain extent, be resisted. If, however, the tone of the uterus be deficient then a greater or less number of these villi may fail to penetrate efficiently the substance of the uterus, and consequently, the walls of the vascular sinuses being deprived of that support which is essential for their well-being, will rupture, it may be, spontaneously, or on the slightest provocation.

The most common cause of hæmorrhage during pregnancy is deficient tone in the structure of the uterus, and in the majority of these cases the hæmorrhage, which is unaccompanied by pain, occurs soon after the vessels of the allantois begin to penetrate the chorionic villi, as early it may be as the fourth, but usually about the fifth or sixth week of pregnancy. The hæmorrhage thus produced may persist, with intermissions, for a greater or less length of time, until even the sixth month of pregnancy, without the process of gestation being thereby interrupted, or the vitality of the fœtus impaired. The blood, under such circumstances is derived from vascular sinuses which have ruptured, and it is evident that the result of this accident will depend upon the extent to which these maternal structures are thus involved. As, however, the breach in the continuity of the walls of these channels is due to the inefficient support which they receive from the opposing chorionic villi, it is probable that the sinuses which are located towards the periphery of the so-called decidua serotina are more likely to be disturbed in this manner than are those which occupy a more central position. This, no doubt, accounts for the ease with which the external hæmorrhage occurs in all these cases.

Before or after the structure known as the placenta has been formed hæmorrhage may be induced by physical shock, and the disturbance thus produced may be the result of direct or indirect violence. In the majority of these cases the premonitory symptoms

* "Quain's Anatomy," Edition 8th, vol. II., p. 706.

of abortion—pain and hæmorrhage—are witnessed very soon after the occurrence of the accident. If the shock is experienced after the eighth week of pregnancy, then the compact union which exists between the chorionic villi and the serotina may prevent the blood thus extravasated from forthwith making its escape externally, and consequently labour-like pains may be complained of for a greater or less length of time before any hæmorrhagic discharge from the uterus makes its appearance. The immediate effect of an accident of this description is not, however, regulated altogether by the extent to which the placental tissue has been disturbed, but depends in some measure upon the state of the uterus itself. The pent-up blood even may become organised, and in undergoing this change it may eventually so interrupt the nutrition of the foetus as to cause its death.

Through the agency of the nervous system hæmorrhage during pregnancy from the serotinal sinuses may be caused by some emotional disturbance, but especially by that arising from intense fear or grief. Under such circumstances the hæmorrhage is usually accompanied by more or less pain, but in all cases the emotional impression will not forthwith exert its deleterious influence upon the pregnant uterus, as it frequently happens that two or even more days may elapse before any evidence of disturbance of this organ is observed.

When a uterus which contains myomatous growths harbours an impregnated ovum the implantation of the chorionic villi may to a greater or less extent be interrupted by the presence of these tumours, and the uterine sinuses which are in this manner deprived of the support of their opposing structures may readily rupture and hæmorrhage may consequently ensue. In a few cases even the blood may flow from a superficial varicose vein which has ruptured, for vessels in this condition are occasionally observed in the unimpregnated state in the neighbourhood of myomatous tumours, which are developing towards the interior of the uterus.

Hæmorrhage may also be caused by uterine contractions excited by pelvic adhesions, which so bind down the uterus that it cannot expand sufficiently or rapidly enough for the developing ovum.

Hæmorrhage may arise, too, in consequence of the presence of a pathological product of conception, such as hydatid mole.

In a few rare cases the blood comes not from the interior of the uterus, but from the cervix, which either shortly before or during pregnancy has become the seat of cancerous change.

*Treatment.*—If the hæmorrhage is due to want of tone in the uterus, a condition which is often caused by over-lactation, too frequent child-bearing, or repeated emotional disturbances, then some drug should be administered which will, as far as possible, maintain the tone of this organ. To effect this small and frequently-repeated doses of ergot and iron or ergot and strychnia may be given. If the hæmorrhage has been induced by physical or mental shock, and is accompanied by pain, then a bromide salt or opium, conium, grindelia, &c., should be tried. Under any circumstances absolute rest should be enjoined, constipation should be avoided, and a non-stimulating but nutritious diet should be allowed.

